# Integration of Sensory Force Feedback Is Disturbed in CRPS-Related Dystonia

**DOI:** 10.1371/journal.pone.0060293

**Published:** 2013-03-26

**Authors:** Winfred Mugge, Frans C. T. van der Helm, Alfred C. Schouten

**Affiliations:** 1 Department of BioMechanical Engineering, Delft University of Technology, Delft, The Netherlands; 2 Department of Physical Therapy and Human Movement Sciences, Northwestern University, Chicago, Illinois, United States of America; 3 Laboratory of Biomechanical Engineering, MIRA Institute for Biomedical Technology and Technical Medicine, University of Twente, Enschede, The Netherlands; Glasgow University, United Kingdom

## Abstract

Complex regional pain syndrome (CRPS) is characterized by pain and disturbed blood flow, temperature regulation and motor control. Approximately 25% of cases develop fixed dystonia. The origin of this movement disorder is poorly understood, although recent insights suggest involvement of disturbed force feedback. Assessment of sensorimotor integration may provide insight into the pathophysiology of fixed dystonia. Sensory weighting is the process of integrating and weighting sensory feedback channels in the central nervous system to improve the state estimate. It was hypothesized that patients with CRPS-related dystonia bias sensory weighting of force and position toward position due to the unreliability of force feedback. The current study provides experimental evidence for dysfunctional sensory integration in fixed dystonia, showing that CRPS-patients with fixed dystonia weight force and position feedback differently than controls do. The study shows reduced force feedback weights in CRPS-patients with fixed dystonia, making it the first to demonstrate disturbed integration of force feedback in fixed dystonia, an important step towards understanding the pathophysiology of fixed dystonia.

## Introduction

Humans use proprioception, vision and the sense of touch to effectively handle objects with a wide range of mechanical properties. Sensory feedback is noisy and has limited accuracy [1], [2]. In the central nervous system the sensory feedback channels are integrated and weighted to improve the state estimate [3], [4]. For example, during balance control, the relative weights of the sensory inputs from the vestibular system, mechanoreceptors and vision [5]–[7] shift with environmental properties, i.e., sensory reweighting [8]. To effectively weight sensory feedback channels, an estimate of their accuracy is required. Bayesian inference has been suggested to underlie sensory weighting [2]. Similar sensory weighting occurs between force and position within the proprioceptive system. Object stiffness, the physical relationship between position and force, allows translation from one modality into the other. When handling stiff objects like a cup, deflections are negligible so position holds no information on the applied force. However, when handling soft objects like a sponge, deflections are large and allow position feedback to contribute to the estimated force and vice versa. When stiffness is known, combining the sensory feedback of these two modalities (sensory integration) provides increased accuracy of the estimate of either force or position [9]. Position feedback is weighted heavier on soft objects (large deflections), while force feedback is weighted heavier on stiff objects (small deflections) in healthy subjects.

Complex Regional Pain Syndrome (CRPS) is characterized by persistent pain, autonomic and trophic features [10]–[12] and is commonly preceded by a minor to severe trauma to a limb in the absence of an obvious nerve lesion [11], [13]. About 25% of the CRPS-patients develop fixed dystonia featuring abnormal postures and sustained muscle contractions, of which the underlying cause is unknown [14]. Dysfunctional sensorimotor integration has been suggested to play a role in the pathogenesis of dystonia [15], [16]. The fact that many forms of focal dystonia can be relieved by “sensory tricks” is a strong indicator that sensory information is an important factor in focal dystonia [17]. In addition, several studies specifically report dysregulation of force in dystonia. Recent modeling studies on the pathophysiology of fixed dystonia support involvement of force dysregulation as the computational neuromuscular model explained all defined features of fixed dystonia only with disturbed force feedback [18], [19]. Experimentally, the force variance during isometric force tasks increased in subjects with childhood dystonia due to cerebral palsy [20]. Moreover, impairment of the ability to rapidly generate force and to voluntarily relax in patients with focal hand dystonia has been suggested to be related to down-regulation of sensory input [21], [22]. Problems to grasp and manipulate objects is a frequently encountered phenomenon in movement disorders [23], [24]. For example, patients with writer's cramp have increased grip force when lifting an object [25], [26]. Grip force adapts with sensory feedback suggesting that inaccurate grip force scaling is a manifestation of impaired sensorimotor integration [27], [28]. To study sensorimotor integration within the proprioceptive system in fixed dystonia we used a target matching paradigm where force and position were related using a (virtual) spring. If indeed sensory force feedback is unreliable, than Bayesian inference would dictate patients with fixed dystonia to reweight force and position feedback, favoring position feedback.

## Materials and Methods

### Subjects

After providing written informed consent, twenty volunteers – 10 CRPS-patients with fixed dystonia and 10 healthy controls – participated in the study that was approved by the medical ethics committee of the Leiden University Medical Center. The controls and patients were matched for age and gender (patients' mean age: 50.3 years (SD 10.3); controls' mean age: 50.8 years (SD 10.5); 1 male and 9 female in each group) and had equal handedness distribution (7 right-handed and 3 left-handed). All patients diagnosed with CRPS and dystonia were recruited in the Leiden University Medical Center (LUMC) and fulfilled the criteria for CRPS-I of the International Association for the Study of Pain (IASP) for at least one upper extremity [13].

### Approach

**Figure 1:**
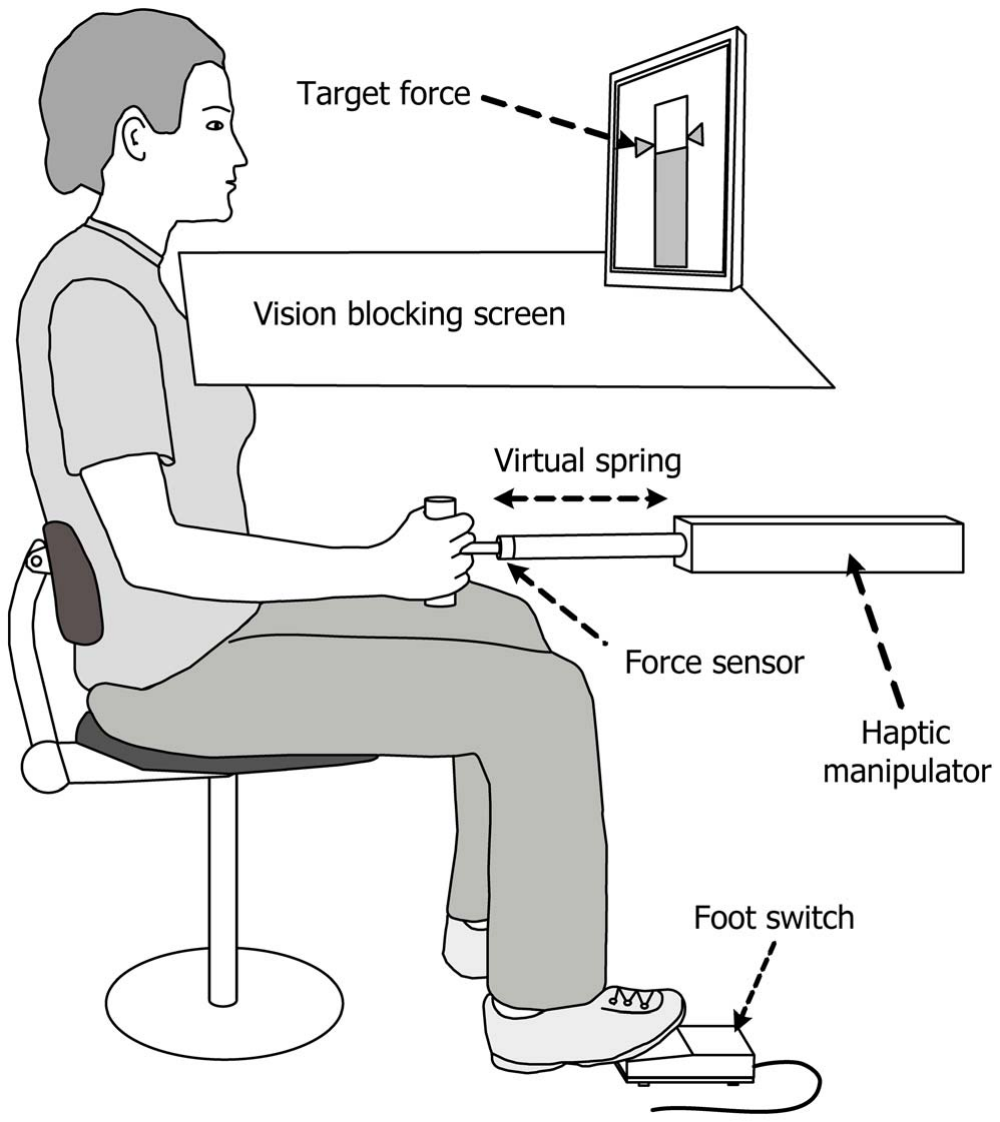
Experimental setup. The subject controlled a haptic manipulator that simulated a spring. During reference trials the measured force was displayed together with the target force. During blind and catch trials the visual feedback was disabled. The subject operated a foot switch to indicate (s)he believed to have acquired the target force.

We used a novel force-matching task that enables quantification of the sensory weighting of force and position [9]. Subjects held the handle of a linear haptic manipulator (Fig. 1) with their dominant hand (controls) or the affected hand (patients; in case of two affected arms the dominant one was used). The arm and handle were blocked from vision to exclude undesired visual feedback. The manipulator simulated a spring and switched between two spring models:

1. linear spring with stiffness 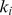
(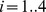): exerting force 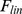, at position 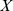, according to Eq. 1:
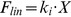
(1)
2. non-linear spring which exerts force *F_target_*+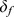 at the position where the linear spring would have exerted the target force 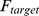:
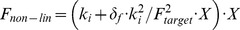
(2)

The experimental protocol, with target force 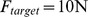 and 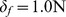, consisted of four blocks of trials with spring stiffnesses (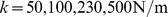). The order of the blocks was randomized and with every new block the subject performed 15 training trials with onscreen visual feedback of the force which enabled the subject to learn the task and familiarize with the stiffness. The spring relates force and position such that they can be integrated by the CNS to get an estimate of either one. The subject was instructed to operate a foot switch when an indicator bar, representing the exerted force, was aligned with the target indicators. Pressing the foot switch triggered a force measurement of 0.6 second at a sample rate of 250 Hz. After each measurement, the subject was instructed on-screen to return to the starting position, i.e., the zero-length of the spring. The next trial was automatically initiated when the subject had crossed the starting position which was indicated by the appearance of the instruction for the next trial. After training, the subject performed a series of trials composed of three trial types:

1. Reference trials where the subject was instructed to apply the indicated force using the onscreen indicators, exactly as in training trials.
2. Blind trials where the subject was instructed to reproduce the trained force blindly and to operate the foot switch when (s)he thought (s)he attained the trained force.
3. Catch trials were blind trials where the linear spring was covertly replaced by the non-linear spring. The spring model was always substituted at the zero-length position, to prevent the subject from noticing any change in force.

Blind/catch trials were alternated with reference trials to prevent drift from the trained force. On average, one in three blind trials was randomly replaced by a catch trial, effectively providing one catch trial every six trials. A total of 12 catch trials were recorded per block.

The difference in force (ΔF) between the blind trials and the catch trials revealed the sensory weighting between force and position feedback. The disparity in the spring environment allows the separate weights of force and position feedback to be determined, because in the catch trials force feedback biases the exerted force toward the trained force and position feedback toward the trained position.

### Data analysis

For every trial, the measured force was averaged over the 0.6-second measurements. To prevent bias to the data due to accidental presses of the foot switch all trials with an average force of less than 5.0N were ignored. Subsequently, the force during reference, blind and catch trials were averaged over the repetitions. ANOVA's were performed to test for an effect of group on the force difference between the blind and the catch trials (ΔF) and the sensory weights. Effects of stiffness on the force exerted during reference, blind and catch trials and on the force difference between the blind and the catch trials (ΔF) as well as on the sensory weights were tested for the CRPS-patients and controls separately. Post hoc tests with Bonferroni-correction for multiple comparisons were performed on trial type.

To compare sensor accuracy, paired t-tests between CRPS-patients and their age and sex-matched controls on the standard deviation of reproduced forces with an infinitely stiff spring, and the standard deviation of reproduced positions with a zero-stiffness spring were performed. An additional paired t-test compared the ratio of the standard deviations because according to maximum likelihood estimation the optimal weighting depends upon this ratio [9].

## Results

**Figure 2:**
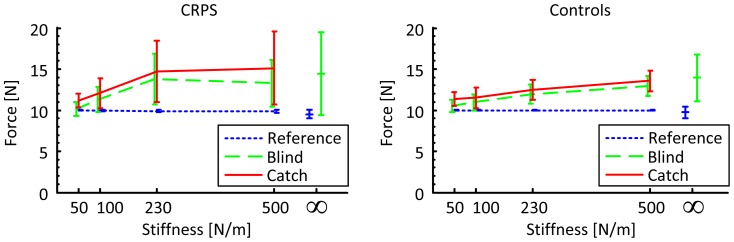
The measured forces in the three trial types against spring stiffness. Mean force with error bars indicating standard deviation in CRPS-patients (left) and in controls (right). For reference, both panels are supplemented with the measured forces in the reference and blind trials with the infinitely stiff spring.

Fig. 2 demonstrates that both controls and CRPS-patients closely approximated the target force of 10N during reference trials with small standard deviations. In the target matching task, reference trials were alternated with blind trials in which the target force was blindly reproduced by loading a linear spring. On occasion a blind trial was covertly replaced by a catch trial with a non-linear spring revealing the sensory weighting between force and position. A significant effect of group was found on the exerted force across all stiffnesses and trial types (p<0.05) as well as an interaction effect of trial type*stiffness (p<0.001). Significant effects of stiffness and trial type on the exerted force were found for both CRPS-patients and controls (all p<0.001). Post hoc analysis revealed that both the CRPS-patients and the controls exerted a higher force during blind trials compared to reference trials, indicating that without visual feedback of the force the subjects underestimated the exerted force (both p<0.001). Additionally, the force during catch trials with the non-linear spring was slightly higher than during the blind trials with the linear spring, which was significant for controls (p<0.01), but due to greater variance was not significant for CRPS-patients (p = 0.278).

**Figure 3:**
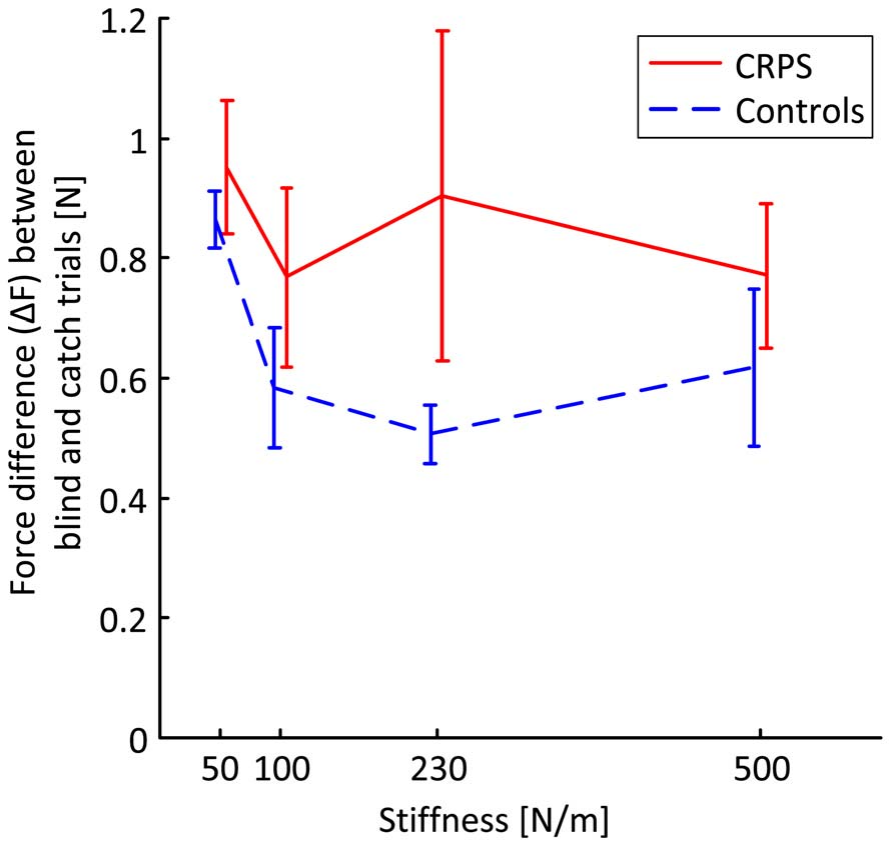
Force difference (ΔF) between the catch and the blind trials. Mean force difference in CRPS-patients (solid) and in controls (dashed) with error bars indicating the standard error of the mean.

Fig. 3 presents the measured difference in force between the blind and the catch trials (ΔF). The results show that the force difference was greater in CRPS-patients with fixed dystonia than in controls (p<0.05). With increasing spring stiffness, controls reduce the force difference indicative of a weight shift toward force feedback (p<0.05), while CRPS patients with fixed dystonia do not (p = 0.903).

**Figure 4:**
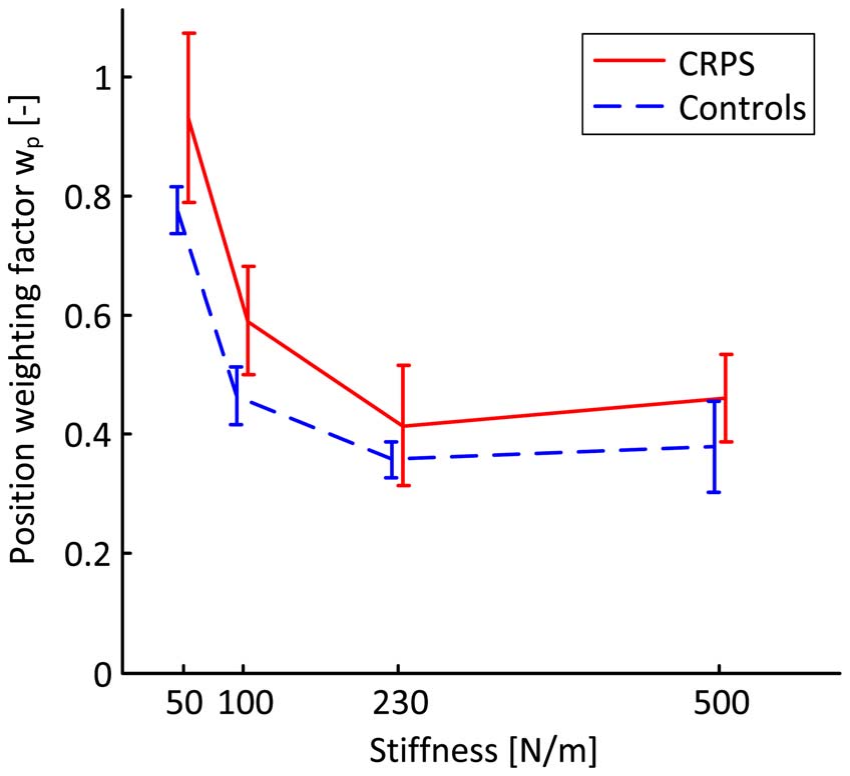
Sensory weighting of position feedback. Mean position weights in CRPS-patients (solid) and in controls (dashed) with error bars indicating the standard error of the mean. Note that 1.0 implies only position weighting, while 0.0 implies only force weighting.

**Figure 5:**
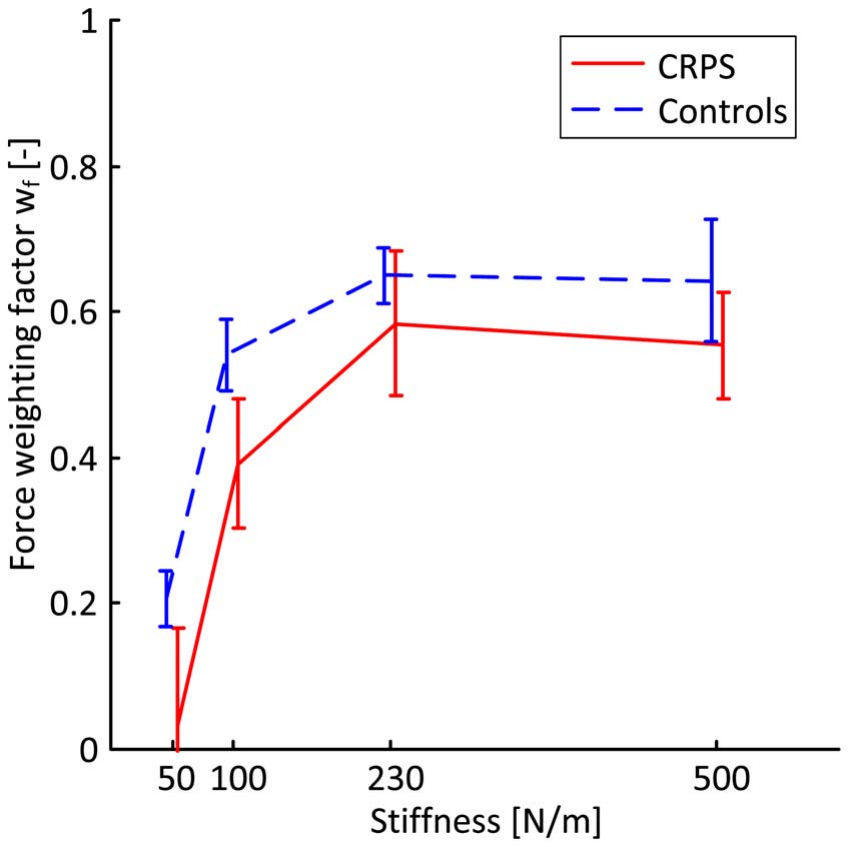
Sensory weighting of force feedback. Mean force weights in CRPS-patients (solid) and in controls (dashed) with error bars indicating the standard error of the mean. Note that 1.0 implies only force weighting, while 0.0 implies only position weighting.

Note that at 10.0N, a force difference (ΔF) of 1.0N indicates that the position was reproduced (position weighting only), while a force difference (ΔF) of 0.0N indicates that the force was reproduced (force weighting only). The patients as well as their age and sex-matched controls produced substantially higher reproduction forces. The force difference between the linear and non-linear spring increases with force level, so to attain the position weighting factor the measured force difference was scaled by the force difference between the linear and non-linear spring at the position as produced in the blind trial. The position weighting factors are presented in Fig. 4. A marginally significant group effect on the position weighting factor was found with higher weighting of position in CRPS-patients with fixed dystonia than in controls (p<0.10). Similarly, the force weighting factor was calculated by scaling the measured position difference between the blind and catch trials by the position difference between the linear and the nonlinear spring at the force produced in the blind trial (Fig. 5). A significant group effect on the force weights was found with a consistently lower weighting of force in CRPS-patients with fixed dystonia than in controls (p<0.05). Apparently, weighting of force and position in CRPS-patients is biased toward position.

The standard deviation for blind force matching (σ_f_) in case of infinite spring stiffness was 1.82N (SD 1.06) for controls and 2.31N (SD 1.61) for patients. The standard deviation for blind position matching (σ_x_) in case of no spring was 9.2mm (SD 2.7) for controls and 11.6mm (SD 4.6) for patients. The ratio σ_f_/σ_x_ was 205N/m (SD 121) for controls and 218N/m (SD 162) for patients. The force and position variability and their ratio were not significantly different between patients and controls (σ_f_: p = 0.488, σ_x_: p = 0.157, and σ_f_/σ_x_: p = 0.855). Given these results the weighting is not expected to be considerably different in CRPS-patients than in controls, according to maximum likelihood estimation [9].

## Discussion

Our findings suggest that CRPS-patients did not optimally weight the sensory inputs. Nevertheless, the CRPS-patients do show adaptation of the sensory weights with stiffness which suggests that sensorimotor integration is not dysfunctional. It seems reasonable to assume that the consistent bias toward position feedback is purposeful, indicative of reduced reliability of force feedback.

CRPS-patients with fixed dystonia produced higher forces in blind and catch trials than controls did, whereas even the controls produced high forces. In contrast to our previous study where the subjects were all male and substantially younger [9], here a scaling of the differences between the blind and catch trials was required to attain the sensory weights. We expect that the higher reproduction forces are due to the age difference, but cannot exclude gender as a factor since 9 out of 10 subjects were female (women are more predisposed to CRPS).

Certain aspects of our study correspond to previous studies on grip force adaptation with task-specific dystonia. Healthy subjects often overestimate the grip force required to lift a novel object and then adapt the force rapidly within the first three lifts [29], [30]. Interestingly, all subjects with task-specific dystonia showed this adaptation, but consistently applied higher force levels than the controls even after ten lifts when no further adaptation occurs [24]. In addition, a previous study [22] showed that patients with dystonia have similar levels of force variability to that of controls at low force levels (25% of maximum voluntary contraction). This corresponds to our finding that force variability of patients was not significantly different from controls at the relatively low target force of 10N that we used.

In a previous study we have shown that in force matching tasks with known stiffness conditions, there is no difference between position and force tasks with respect to sensory weighting [9]. To prevent unnecessary strain to the CRPS-patients in the current study only the force task was performed. Instructing a subject to reproduce either force or position can be interpreted as focusing the subject's attention to one of the two modalities, possibly weighting force feedback heavier during a force task and position feedback during a position task. Although literature has shown that attentional manipulation of a specific sensory modality does not influence the relative weighting of that modality [31], the potential bias due to the current instruction would be toward force feedback and not toward position feedback.

Here we show that CRPS-patients with fixed dystonia present significantly different reproduction forces and sensory weighting that is biased toward position feedback. Assessment of sensorimotor integration may provide insight into the pathophysiology of fixed dystonia. The current findings support involvement of disturbed force feedback in fixed dystonia.
